# Schistosomiasis Burden and Its Association With Lower Measles Vaccine Responses in School Children From Rural Cameroon

**DOI:** 10.3389/fimmu.2018.02295

**Published:** 2018-10-09

**Authors:** Justin Komguep Nono, Severin Donald Kamdem, Palmer Masumbe Netongo, Smritee Dabee, Michael Schomaker, Alim Oumarou, Frank Brombacher, Roger Moyou-Somo

**Affiliations:** ^1^The Medical Research Centre, Institute of Medical Research and Medicinal Plant Studies, Ministry of Scientific Research and Innovation, Yaoundé, Cameroon; ^2^Division of Immunology, Health Science Faculty, University of Cape Town, Cape Town, South Africa; ^3^Cytokines and Diseases Group, International Centre for Genetic Engineering and Biotechnology, Cape Town, South Africa; ^4^Immunology of Infectious Disease Research Unit, South African Medical Research Council, Cape Town, South Africa; ^5^Department of Biochemistry, University of Yaoundé I, Yaoundé, Cameroon; ^6^Division of Medical Virology, Department of Pathology, University of Cape Town, Cape Town, South Africa; ^7^Centre for Infectious Disease Epidemiology & Research, University of Cape Town, Cape Town, South Africa; ^8^District Hospital of Mfou, Yaoundé, Cameroon; ^9^Wellcome Centre for Infectious Diseases Research in Africa, Institute of Infectious Disease and Molecular Medicine, Faculty of Health Sciences, University of Cape Town, Cape Town, South Africa

**Keywords:** schistosomiasis, Cameroon, risk factors, infection, fibrosis, vaccine responses, measles

## Abstract

**Background and Methods:** Schistosomiasis is debilitating and reported to impair immune responsiveness of infected hosts. In Cameroon, mass drug administration (MDA) is used in schoolchildren to reduce transmission of *S. haematobium* and *S. mansoni*. The effects of MDA and the impact of schistosomiasis on the titers of antibodies in vaccinated children have been poorly studied. We therefore assessed the prevalence of schistosomiasis in schoolchildren, eight months after MDA, in two locations: Barombi Koto (BK), endemic for *S. haematobium* (*N* = 169) and Yoro (Y), endemic for *S. mansoni* (*N* = 356). Age, gender, residence time and frequency of contact with river water were assessed as risk factors for infection and morbidity in both localities. In 70 schoolchildren from BK and 83 from Y, ultrasound was used to assess morbidity according to the WHO guidelines. Evaluation of measles antibodies was performed in previously vaccinated schoolchildren (14 with *S. haematobium* and 12 egg-negative controls from BK and 47 with *S. mansoni* and12 egg-negative controls from Y).

**Principal Findings and conclusions:** The prevalence of *S. haematobium* was 25. 4% in BK (43/169) and 34.8% for *S. mansoni* in Y (124/356), indicating the persistent transmission of schistosomiasis despite MDA. Older age (AOR 1.31; 95%CI 1.12–1.54) and higher frequencies of exposure to river water (AOR 1.99; 95%CI 1.03–3.86) were identified as risks for infection in BK whereas only older age (OR 1.15; 95%CI 1.04–1.27) was a risk for infection in Y. Bladder pathology (score 2 to 5) was observed in 29.2% (7/24) of egg-positive children in BK and liver pathology (pattern C) in 31.1% (19/61) of egg-positive children in Y. There was a positive correlation between *S. haematobium* egg burden and bladder pathology (AOR 1.01; 95% CI 0.99–1.02) and positive correlation between *S. mansoni*-driven liver pathology and female gender (AOR 3.01; 95% CI 0.88–10.26). Anti-measles antibodies in vaccinated children were significantly lower in *S. mansoni*-infected when compared to egg-negative controls (*p* = 0.001), which was not observed in the *S. haematobium*-infected group from BK. Our results demonstrate a questionable efficacy of MDA alone in halting schistosomiasis transmission and confirm a possible immunomodulatory effect of *S. mansoni* on response to vaccines.

## Summary

Close to 50 years after the discovery and establishment of praziquantel as an anti-parasitic drug to treat human schistosomiasis, the disease persists and is still a public health concern for hundreds of millions of individuals worldwide. Although the wide implementation of mass drug administration campaigns in endemic areas has considerably helped to reduce the prevalence and burden of the disease encouraging a change of narrative from infection control to elimination, the lack of regular monitoring studies in endemic areas has crippled the development of adequately tailored strategies to ensure the site-specific interruption of the disease transmission. Here, we screened 525 schoolchildren including the most affected age groups in two sites in rural Cameroon, a country of close to 20 million inhabitants with 2 million infected individuals and more than 5 million living in rural areas infested with *S. mansoni* and *S. haematobium* causing hepatointestinal and urogenital schistosomiasis, respectively. In the studied sites, praziquantel has been administered for the past decade once annually to schoolchildren via a well sustained national control program. We found that just 8 months following the last mass drug treatment, prevalence of infection persisted at alarming levels with older children being more at risk of infection, unveiling the inadequacy of limiting drug treatment to young schoolchildren rather than including the whole community with older individuals. Abnormalities of the urinary bladder were more severe when compared to liver lesions arguing for a more frequent regimen of drug administration on the *S. haematobium* site in particular. Females presented with a higher risk of infection in the *S. mansoni* site while contact with the surrounding river waters favored infection at both sites. Overall, a need for further education of the population on the debilitating risk of poor hygienic practices and contact with infested river was identified. Intriguingly, analyses on the *S. mansoni* site revealed a negative association of schistosomiasis on measles vaccine elicited-responses, further reinstating the morbid nature of schistosomiasis on our communities. Our survey, while appraising the extent of disease distribution and impact in two endemic areas also provides guidelines to ameliorate the fight against schistosomiasis, gearing towards more informed approaches for the elimination and not just the control of the disease.

## Introduction

Previous mapping studies have defined that more than two million people have schistosomiasis infection in Cameroon and an additional five million live in high transmission areas within the country (1), a country with a population estimate of just over 20 million people (1). Annual mass drug administration (MDA) of praziquantel to schoolchildren in endemic areas represents the country primary control strategy against the disease (2). The country is home to *S. mansoni* (3–7) and *S. haematobium* (1, 3, 4, 8) causing the hepatointestinal and urogenital forms of the disease respectively.

Overall, the prevalence of schistosomiasis in Cameroon in particular and Africa in general has been reduced over the last decades as a result of mapping of endemic areas and sustained MDA campaigns with praziquantel (9). This has not been sufficient in interrupting the transmission cycle of the parasite and eliminate the disease. Elimination clearly requires more adequate efforts to complement MDA campaigns as we now realize that MDA alone might just be a stopgap measure (9, 10, 11). In addition to sustained MDA campaigns, long term planning, intersectoral government coordination, health education and improved sanitation are required, on the long term for the elimination of schistosomiasis (2, 9–11).

Generally MDA campaigns are performed annually in endemic areas like Cameroon which did considerably reduce the egg-patent prevalence of the disease at first, but poorly so after several rounds of localized MDA campaigns (5–7). This reported plateau of the disease prevalence further argues for the inadequacy of MDA campaigns alone (9, 11), as presently implemented, to eliminate schistosomiasis and the burden associated with the disease in an endemic site (9, 12). Clearly, in-depth screenings of endemic areas for determinants of persistent transmission and burden are needed in countries like Cameroon (2, 9, 12).

The mapping of the disease prevalence and associated burden has reliably guided anti-schistosomiasis intervention strategies during the control phase of the national anti-schistosomiasis programs (13–15). Defining the prevalence of infection and disease and the biosocial factors that foster the infection and the disease have considerably helped MDA campaigns (1, 2, 5–8, 13, 14). At present, schistosomiasis elimination in endemic areas like Cameroon requires tractable approaches for the mapping of risks of prevalence and burden as this will considerably help ameliorate the current control strategies into elimination tools (9, 11, 12, 14, 16).

Utzinger's and Raso's groups (16–18) elegantly reported on the reduction of schistosomiasis transmission in regions of northern and central Cote d'Ivoire where the MDA campaigns were complemented with health education and community-led pro-sanitary practices tailored to these sites. These studies had initially mapped the determinants of schistosomiasis prevalence and disease in the targeted regions of Cote d'Ivoire (17) revealing a critical lack on the means of stopping schistosomiasis transmission and the overlooked community-led sanitary habits that might help prevent transmission. Upon implementation of health education campaigns and community-led pro-sanitary measures to correct the identified gaps (16), the prevalence and burden of schistosomiasis dropped considerably compared to MDA campaigns alone. This illustrates the added value of regular mapping of endemic areas to identify potential complements for MDA campaigns to aid, not only in controlling, but possibly in totally eliminating schistosomiasis.

Moreover, with numerous reports on outbreaks of preventable diseases in parasite-infested areas, a concern for the presence of a highly immunomodulatory disease such as schistosomiasis on the efficiency of vaccination campaigns is suggested (19–21) but still warrants further investigation. In Cameroon, measles vaccination coverage is still at a sub-standard level with an overall 80% coverage rate. Several measles outbreaks have been reported in 2015 (Center, Far North and North-West regions particularly) and in 2016 (Adamawa, Far North, North and South-West regions) (22) which are all schistosomiasis-diseased areas (1, 2, 3, 5–7, 23). Intriguingly, a recent study in western Kenya elegantly described a crippling effect of mothers diseased with schistosomiasis during pregnancy on their children responsiveness to measles vaccination (19). We therefore hypothesize for a negative role of schistosomiasis on measles vaccine responses that, if proven real, could further impair the already sub-optimal attempts of the national vaccination campaigns against measles.

In our present study, we enrolled, questioned and screened 525 schoolchildren from two sites of rural Cameroon endemic, Barombi Kotto (BK) and Yoro (Y), preferentially endemic for *S. haematobium* and *S. mansoni*, respectively. We determined the egg-patency in the populations 8 months following MDA and defined the risk factors and clinical signs of infection and morbidity. We show that annual MDA campaigns are minimally effective in halting the disease transmission in both sites. We denote the differential influence of biosocial determinants in predisposing to schistosomiasis infection and morbidity. Specifically, we report on the strong tissue destructive nature of urogenital schistosomiasis; we present a positive correlation between the burdens of *S. haematobium* eggs and bladder morbidity on one of our study sites whereas *S. mansoni* egg burden did not appear to be associated with the development of liver pathology on another site. We finally report on the negative association of hepatointestinal schistosomiasis with anti-measles vaccine responses. Overall, a framework for a tailored intervention in two sites endemic for schistosomiasis in rural Cameroon and a case for the impairment of measles vaccination by schistosomiasis are provided.

## Materials and methods

### Ethics statement

The Ministry of Public Health of Cameroon through the National Ethics committee for Human Health Research granted ethical approval for this study (No 2017/03/881/CE/CNERSH/SP). Children and legal guardians were informed on the scope of the study by a physician assisted by school teachers. At each school, written informed assents and consents were given by legal guardians and pupils. All data obtained and herein reported were treated anonymously by the investigators. All schools involved in the study, as well as other schools from the areas were subsequently treated by the National control program for schistosomiasis irrespectively of their parasitological status.

### Study areas and populations

The data were collected in May 2017 in the village of Barombi Kotto (*n* = 169) and the village of Yoro (*n* = 356). The survey took place in two schools in Barombi Kotto (Primary and secondary school of Barombi Kotto) and two schools in Yoro (Public school of Yoro I and public school of Yoro II) surrounding the infested rivers in the villages. As the risk factors of infection and morbidity following MDA had never been determined previously in these sites, sample size was not pre-determined and was dependent on schoolchildren (and legal guardian) willingness to participate. A subsample of the enrolled children, which came back for ultrasonography (70 from BK and 83 from Y), was further screened for bladder morbidity in Barombi Kotto and liver morbidity in Yoro. The details on the study areas have been previously described (1, 3, 23, 24).

### Interview questionnaire

Each child was interviewed by a member of staff of our team, assisted by the class teacher and the legal guardian. The questionnaire recorded demographic data (age, sex), social habits (frequency of encounters with surrounding river) and general self-reported or legal guardian indicated health conditions (rectal pain, frequent tiredness, diarrhea, abdominal pain, vomiting, any pain, vaccination status) and is available upon request.

### Parasitological and morbidity survey

In Barombi Kotto, gross hematuria was assessed visually from the presence or absence of red/brown coloration. From samples of urine (single urine sample per child), 10 ml were syringed through a filter for detection of *S haematobium* eggs, as previously described (6, 23, 25). In Yoro, feces samples were collected from each participant for microscopic analysis (single stool sample per child). Per feces sample, two Kato-Katz slides of 25 mg fecal material each were prepared and microscopically examined for *S. mansoni* species as previously described (3).

### Ultrasound

In Barombi Kotto, a clinician performed ultrasound assessments of the bladder, ureters and kidneys on children and urinary bladder morbidity scores was determined. In Yoro, abdominal echographic scans were performed on participants. In both sites, participants were examined using a portable ultrasonography with transducer of 3.5 MHz. All examinations were performed by the same clinician who was unaware of the infection status of the examined participants. Morbidity scores were defined according to the WHO protocol on the assessment and quantification of schistosomiasis morbidity as recently described (25). Ultrasound examination was complemented by serum rapid diagnostic tests of tissue debilitating diseases and individuals with diseases likely to affect tissue morbidity (hepatitis, HIV) were excluded using Diaspot HBsAg / HCVAb (manufactured by DiaSpot Diagnostics, USA) and Alere HIV RDT (from Abbott Diagnostic, USA), for hepatitis and HIV respectively. Positives cases (2 for Hepatitis B and 3 for hepatitis C) were referred to the local health Centre for follow-up.

### Anti-measles IgG ELISA

Serum samples from examined participants vaccinated against measles, were prepared from whole blood aseptically drawn by venipuncture inside tubes without anti-coagulant. After clotting and centrifugation at 3,500 rpm during 5 mins, serum was separated in new 2.0 ml cryotubes and frozen at −20°C until analyses.

Total anti-measles IgGs was probed by sandwich ELISA using a commercial kit (Demeditec Diagnostics GmbH, Kiel, Germany) as per the manufacturer's instructions with a threshold for positivity at 12 U/ml. Two Cut-Off standards, 2 negative controls and 2 positive controls, provided with the commercial kit, were included during the assay for internal quality control as per the manufacturer's instructions.

### Statistical analyses

Statistical analyses were conducted using GraphPad Prism 6.0 software (http://www.prism-software.com) and STATA version 13 (StataCorp, College Station, TX, USA). Fisher's exact test compared proportions; the non-parametric Mann-Whitney *U* test was used to compare sample medians (26). Descriptive measures (mean, geometric means, standards deviation, frequencies, and percentages) were used to summarize data. Sensitivity, specificity, 95% confidence intervals and *p*-values assessed the predictive accuracy of differences and lack thereof). Univariate and multivariate analyses were carried out using logistic regression or Spearman correlations to assess for possible statistical associations. Adjusted odds ratios (AOR) were reported for variables and 95% CI and *p*-values were determined. A *p*-value <0.05 was considered indicative of statistical significance.

## Results

### Characteristics of the recruited children

**Figure 1:**
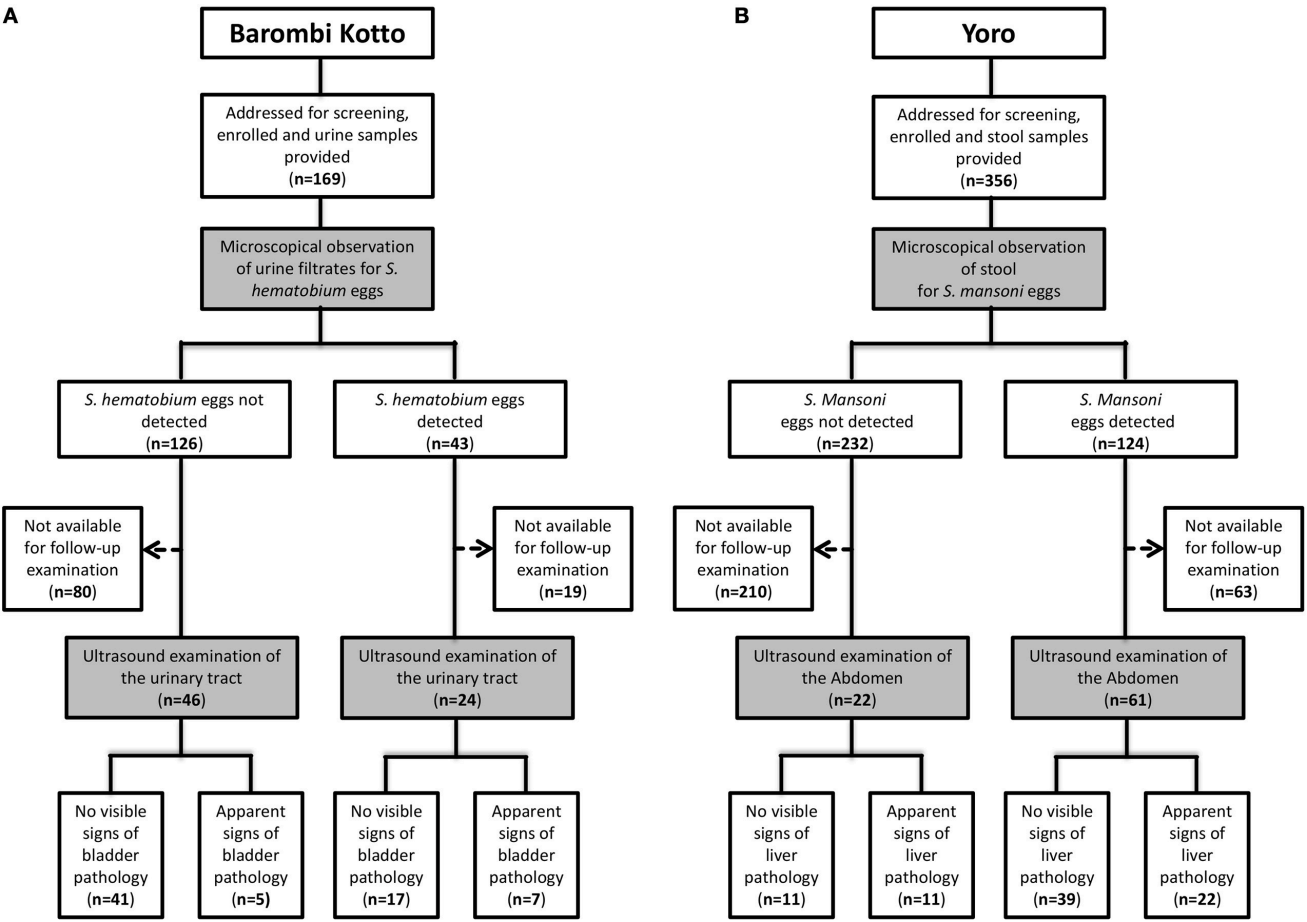
Study flow diagrams. **(A)**. Barombi-Kotto. **(B)**. Yoro.

Complete parasitological data and answers to questionnaires were obtained from 525 schoolchildren (Figure 1 and Table 1) from two endemic sites in rural Cameroon. This included 169 schoolchildren from the *S. haematobium*-infested site of Barombi-Kotto (BK; Mean age of 11.2 years, range 6–20 years) and 356 school-children from the *S. mansoni*-infested site of Yoro (Y; Mean age of 9.5 years, range 4–16 years). Gender distribution of the recruited children was differentially skewed in the two studied sites (40% males in BK and 57% males in Y; *p* < 0.0001, Table 1). All recruited children had received their last praziquantel treatment 8 months prior to enrolment. Children from BK had resided in the endemic site significantly longer than children from Y (mean residence time of 8.6 vs. 6.9 years in BK and Y, respectively; *p* < 0.0001). The residence time was determined by our questionnaire and was defined as the number of years the participant spent in the endemic areas (BK or Y at large) and was validated by the legal guardians before consideration. Overall, a considerable percentage of children enrolled had lived their entire lives in the studied areas with no migration out of the endemic area (93/169 in BK and 166/356 in Y).

**Table 1:** Schistosomiasis prevalence in recruited children around Barombi Kotto Lake and Yoro village 8 months after MDA. Distribution of children by age groups and infection status.

**Age groups**	**Around barombi kotto lake***** S. hematobium***** (*****N*** = **169)**		**Yoro village *****S. mansoni***** (*****N*** = **356)**	
	**Negative *N* (%)**	**Positive *N* (%)**	**Negative *N* (%)**	**Positive *N* (%)**
All cases	126 (74.6)	43 (25.4)	232 (65.2)	124 (34.8)
<10 years	37 (97.4)	1 (2.6)	128 (74)	45 (26)
(10–14) years	77 (70.6)	32 (29.4)	100 (57.8)	73 (42.2)
>14 years	12 (55.5)	10 (45.5)	4 (40)	6 (60)

Egg screening in the children excreta (urine for BK and stool for Y) revealed that 43 children (25.4%) from BK excreted *S. haematobium* eggs in their urines whereas 124 children (34.8%) from Y excreted *S. mansoni* eggs in their stools (Table 1).

**Figure 2:**
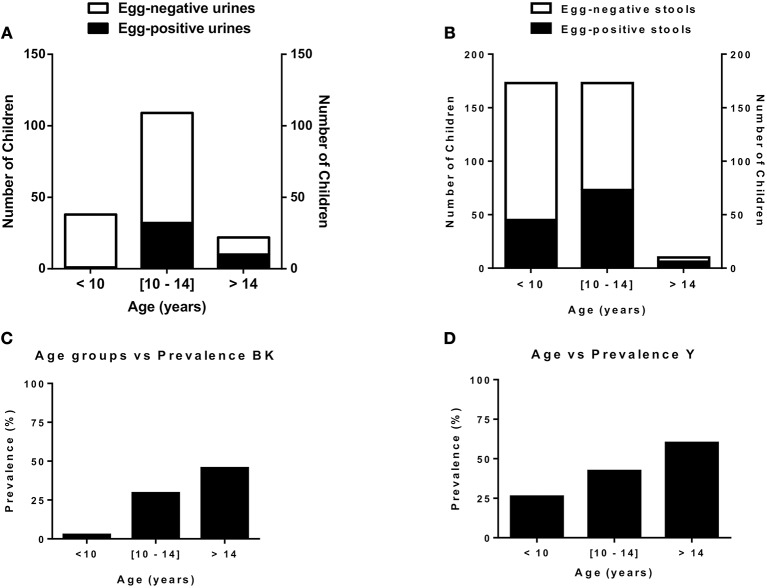
Age distribution & schistosomiasis prevalence in the two endemic communities studied. **(A)**. Distribution of children by infection status and age in Barombi-Kotto. **(B)**. Distribution of children by infection status and age in Yoro. **(C)**. Prevalence of infected children in Barombi-Kotto. **(D)**. Prevalence of infected children in Yoro.

The most abundant cases of egg positivity in excreta were found in children aged 10–16 (mode at 11 years old) in BK and 7–13 in Y (mode at 12 years old) even though prevalence of egg positive excreta kept increasing with age in both sites (Figure 2).

### Risk factors of infection

Multivariable logistic regression analyses showed that age was associated with infection in both areas (AOR 1.32; 95% CI 1.13–1.54 in BK and AOR 1.15; 95% CI 1.04–1.27 in Y) whereas contact with the surrounding water (AOR 2; 95% CI 1.03–3.9) and gender (AOR 2; 95% CI 1.03–3.86) were associated with infection only in children from BK (summarized in Table 2).

**Table 2:** Risks factors for schistosomiasis infection in schoolchildren around Barombi kotto and Yoro river.

**Risk factors**	**Around barombi kotto** ***S. hematobium*** **(*****N*** = **169)**			**Yoro village** ***S. mansoni*** **(*****N*** = **356)**		
	**Negative**	**Positive**	**AOR (95% CI)**	**Negative**	**Positive**	**AOR (95% CI)**
Age in years (mode (range))	10 (6–20)	11 (8–20)	1.32 (1.13–1.54)**	9 (4–16)	12 (4–16)	1.15 (1.04–1.27)**
**SEX**						
Male	55	13	Reference	137	67	Reference
Female	71	30	2.33 (1.01–5.39)*	95	57	1.29 (0.82–2.04)^NS^
Length of residence in years (mode (range))	10 (1–18)	10,11 (2–20)	1.03 (0.93–1.15)^NS^	9 (0.25–16)	10 (0.083–15)	1.03 (0.97–1.1)^NS^
Frequency of contact with river water/day (mode (range))	2 (1–3)	2 (1–5)	2 (1.03–3.9)*	1 (0–3)	1 (0–3)	0.86 (0.66–1.14)^NS^

AOR, Adjusted Odds ratio; 95% CI, 95% Confidence Interval; ^NS^p >0.1;

* p < 0.05;

** *p < 0.01*.

### Clinical correlates of infection

Clinical signs were analyzed to determine their suitability as symptoms of the diseases in our two study sites. In BK, 22/43 (51.2%) of infected children presented gross hematuria, that was present only in 6/126 (4.8%) of egg-negative children (sensitivity of 51.2%, specificity of 95.2% and positive predictive value of 78.5% for gross hematuria in predicting *S. haematobium* infection in BK). In Y village, abdominal pain, diarrhea, recurrent tiredness, and rectal pain occurred with low positive predictive values individually (ranging from 28.1 to 30.7%) or in combination (positive predictive value of 31.4%) with relation to *S. mansoni* infections.

### Burden of infection: egg burden

Overall, the burden of eggs (Table 3) in the urines of egg-positive children from BK was heavy in average with a geometric mean of 78 eggs per 10 ml of urine (95% CI 62.9–96.6) whereas the egg burden in Y was light in average with a geometric mean of 82 eggs per g of stool (95% CI 68.5–97.6). In Y, females were more represented in higher egg burden categories than males (*X*^2^ = 6.6; *p* = 0.036).

**Table 3:** Infection intensity in all egg-positive children recruited from Barombi Kotto and around Yoro river.

**Egg burden**	**Around barombi kotto** ***S. haematobium* (*N* = 43)**	**Yoro village** ***S. mansoni* (N = 124)**
**NUMBER OF EGGS**^#^ **[GM (95% CI)]**		
All cases	78 (62.9–96.6)	81.7 (68.5–97.6)
Male	67.3 (47.1–96.1)	71.2 (58.3–86.9)
Female	83.1 (63.1–109.5)	96.1 (70.4–131.4)
**CLASS OF PARASITE LOAD**^##^ **[N (%)]**		
**Light**	**(**<**50 eggs/10 ml urine)**	**(**<**100 eggs/g feces)**
All cases	13 (30)	75 (60.5)
Male	5	47
Female	8	28
**Moderate**	**(N/A)**	**([100–400[eggs/g feces)**
All cases	N/A	41 (33.1)
Male	N/A	17
Female	N/A	24
**Heavy**	**(**≥**50 eggs/10 ml urine)**	**(**≥**400 eggs/g feces)**
All cases	30 (70)	8 (6.4)
Male	8	3
Female	22	5

GM, Geometric mean; 95% CI, 95% Confidence Interval;

# Mann-Whitney test, male x female p = 0.44 in BK and p = 0.21 in Y;

## *Chi-square or Fishers exact test, male × female, p = 0.44 in BK and p = 0.036 in Y*.

### Tissue morbidity

Ultrasound data were collected from 70 children (mean age 12 year, range 9–15 year) in Barombi-Kotto lake and from 78 children (mean age 10 year, range 7–12 year) in Yoro village.

*S. haematobium*-specific bladder morbidity was observed in 17.1% (12/70) of the examined children in BK (Table 4). Most common lesions concerned wall thickening (*n* = 18), wall irregularity (*n* = 59) and polyps (*n* = 4). In children with higher bladder morbidity, gross hematuria was more frequent, and the mean geometric egg burden was higher (Table 4).

**Table 4:** Urinary bladder morbidity in schoolchildren from BK according to gross hematuria and presence or absence of *S. haematobium* eggs in urine.

**Urinary bladder score**	**with gross hematuria**	**Egg-negative**	**Egg-positive**	**Egg burden**
	**(*N* = 19)**	**(*N* = 46)**	**(*N* = 24)**	GM (95% CI)*
0–1^#^	13 (68.4)	41 (89.1)	17 (70.8)	73.7 (53.2–102.1)
= 2^##^	3 (15.8)	5 (10.9)	3 (12.5)	81.1 (21.8–301.8)
≥3^##^	3 (15.8)	0 (0)	4 (16.7)	163.6 (45.6–587.1)

* *GM, Geometric Mean of egg burden per 10 ml urine calculated for microscopically S. haematobium-positive individuals only*.

# No lesions;

## *Lesions suspected/present*.

*S. mansoni*-specific liver lesions were present in 36% (30/83) of the examined children in Y (Table 5). Liver image patterns were from A to C, with the most affected of the examined children in Y classified as C (30/83), indicative of echogenic livers with possible fibrosis.

**Table 5:** *Schistosoma mansoni*-associated liver image pattern and *S. mansoni* infection in children examined (ultrasonography) in Yoro village.

**Liver image pattern**	**Egg-negative**	**Egg-positive**	**Egg burden**
	**(*N* = 22)**	**(*N* = 61)**	GM (95%CI)*
A^#^	10 (45.5)	33 (54.1)	100.4 (70.6–142.8)
B^#^	1 (4.5)	4 (6.6)	51.4 (13.5–195.3)
C^##^	11 (50)	19 (31.1)	147.7 (77.1–283.1)

* *GM, Geometric Mean of egg burden per gram of feces calculated for microscopically S. mansoni-positive individuals only*.

# No lesions;

## *Lesions possible*.

### Risk factors of tissue morbidity

Multivariable logistic regression analyses showed egg burden to positively associate, (AOR = 1.016; CI = 0.999–1.003) with the onset of bladder morbidity in BK whereas a risk was associated to females in Y (AOR = 3.02; CI = 0.89–10.3). Intriguingly, we could not show that age constitutes a risk factor for tissue morbidity in our examined study participants from both sites, nor did the length of residence in the endemic areas.

### Measles vaccine responses

**Figure 3:**
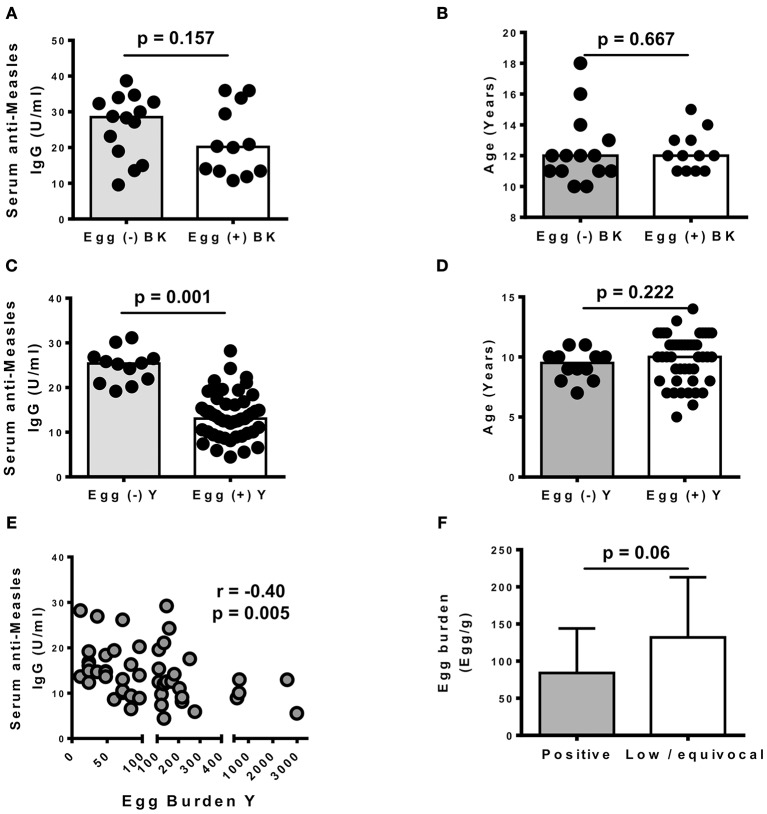
Effect of Schistosomiasis infections on vaccine-induced anti-measles responses in schoolchildren from Barombi Kotto and around Yoro river. **(A)** Influence of *S. haematobium* (BK) infection on serum anti-measles IgG levels. *N* = 26 with 14 egg (–) and 12 egg (+) **(B)** Age distribution in children tested for vaccine responses in BK. **(C)** Influence of *S. mansoni* (Y) infection on serum anti-measles IgG levels. *N* = 59 with 12 egg (–) and 47 egg (+). **(D)**. Age distribution in children tested for vaccine responses in Y. **(E)** Correlation between *S. mansoni* egg burdens and serum anti-measles IgG levels in Yoro. **(F)** Egg burdens and anti-measles antibody titers in examined children from Yoro. Low / equivocal (< 12 U/ml); positive titers (≥12 U/ml). Bars represent the medians and vertical lines represent the interquartile ranges.

To determine the effect of schistosomiasis on measles vaccine responses in our sites, serum levels of anti-measles IgG were determined in a subset of previously vaccinated children from our study sites. The latter included 26 children in BK (14 egg-negative and 12 egg-positive) and 59 children in Y (12 egg-negative and 47 egg-positive). Vaccination cards were requested from all children's parents/guardians and all participants included for this part of the study were vaccinated at 9 months of age as recommended by the Cameroon extended program of vaccination using the live attenuated measles vaccine manufactured by Serum Institute of India LTD. We noted that *S. haematobium* infection did not significantly associate with altered anti-measles IgG antibodies levels in the serum of vaccinated children in BK (*p* = 0.157, Figure 3A). We also confirmed a similar age distribution between egg-negative and egg-positive children from BK (Figure 3B), ruling out a confounding effect from the age between both groups. Intriguingly, however, previously vaccinated children presenting an egg-patent *S. mansoni* infection in Y had significantly lower levels of anti-measles (*p* = 0.001) IgG antibodies in their serum (Figure 3C) and this did not associate with a differential age distribution between egg-negative and egg-positive children whose samples were used in this assay (Figure 3D). As shown in Figure 3E, we further noted that these lower IgG levels in the serum of *S. mansoni*-infected children from Y negatively correlated with stool egg burdens (Spearman *r* = −0.4; *p* = 0.005). Importantly, a trend for higher egg burden was observed in Y children with low or equivocal production of IgG antibodies against measles (titers < 12 U/ml) when compared to children with clearly positive antibodies titers (titers ≥ 12 U/ml) (Figure 3F), as defined by the ELISA kit manufacturer (Demeditec Diagnostics GmbH, Kiel, Germany).

## Discussion

Our study further characterizes the burden of schistosomiasis in rural Cameroon, particularly in the *S. haematobium* infested area of Barombi Kotto (1, 23, 24) and the *S. mansoni* infested area of Yoro (3). Our investigations focused predominantly on affected schoolchildren in the areas and report on the infection burdens, the risks of infection, the tissue morbidity profiles and the effect of infection on measles vaccine-elicited serum antibody responses.

Although being subjected to MDA interventions currently operating at treatment cycles of single annual provision, schoolchildren enrolled at both studied sites had a considerable level of infection with schistosomiasis (>20%) just 8 months after last treatment. This, according to WHO programmatic guidelines (27), still warrants regular MDA campaigns on these sites in rural Cameroon. The need for such MDA campaigns is further strengthened by the limitation of our parasitological assessment approach in this study which relied on the observation of a single sample of excreta (stool or urine) from the children. Such an approach will clearly miss lightly infected children where discontinued elimination of eggs in the excreta is rather common, pointing overall to a higher prevalence of infected children than presently defined in our study sites. From initial appraisals reporting prevalence as high as 76% in Barombi Kotto (23), the current report of the egg-positive prevalence of 25.4% in excreta from schoolchildren of this area is encouraging and denotes an effective progress of the National Control program. In Yoro, however, the situation is different with a prevalence of 19% for egg-positive stools in 2001 (3) to a current prevalence of 34.8% in schoolchildren of the site, the village appears to be a “dramatically persistent hotspot” of schistosomiasis (28). In fact, the sustained prevalence of schistosomiasis amid regular MDA is far from uncommon as recently observed in rural Kenya (29). This clearly suggests the inadequacy of current schemes of single annual MDA in endemic regions such as Yoro and warrants a more aggressive approach on such sites. Importantly, a better appraisal of risk factors of infection on such sites remains paramount.

Risk factors of infection (reinfection and/or persistent infection) in these sites were similar to those previously reported (1, 8, 17, 25) i.e. age, gender, frequency of contact with infested water (25, 30–32). We also confirmed a link between hematuria and *S. haematobium* infection on the Barombi Kotto site whereby children with hematuria were more likely to be egg-positive than egg-negative (50 vs. 5%, respectively). This is consistent with several previous studies that have unequivocally established the association between *S. haematobium* infection and hematuria (1, 8, 17, 25). Surprisingly, however, clinical signs usually associated with hepatointestinal schistosomiasis (17, 25) such as abdominal pain, diarrhea, tiredness or rectal pain did not reliably associate with *S. mansoni* infection in Yoro. Clearly, these symptoms are not particular to *S. mansoni* infection. As such, and considering the limited stringency of our study in excluding children with other conditions (bacterial infections, infections with *salmonella spp.*, hemorrhoids or malnutrition for example), and the small sample size of our enrolled schoolchildren, the present distribution of these observed symptoms could well reflect other underlying infections in these children and/or a lack of power. A complementary explanation could be the relatively short span of *S. mansoni* infection these children might have undergone just 8 months after the last MDA on the site in the context of reinfection, rather than persistent infection. This would support the absence of symptoms in recently infected children. In fact, our stool analyses revealed a light burden of infection in most schoolchildren from Yoro arguing in favor of our explanation. It would therefore be desirable to screen for these clinical indicators in the whole community, including those excluded from the MDA programs and more likely to present with heavy infections, to comprehensively address their potential as tools for clinical diagnosis of hepatointestinal schistosomiasis in Yoro.

We also noted that the presence *S. haematobium* eggs strongly translated into pathology as evidenced by a significant association between the egg burden and bladder pathology in Barombi Kotto. However, schoolchildren from Yoro presented with minimal pathology and egg burdens with an absence of association between *S. mansoni* egg burden and liver lesions. This either results from the limited sample size of children enrolled at our sites or could simply further supports the idea of a differential immunogenicity of *S. mansoni* and *S. haematobium* eggs (25, 33) and therefore strongly argues for a parasite-independent regulation of liver lesions during *S. mansoni* infection. In fact, for the latter, our study identified the female gender as a risk factor of liver pathology during *S. mansoni* in Yoro. This is in agreement with previous clinical studies on *S. mansoni* (25, 34). The implication for schistosomiasis control could be considerable if validated in larger studies. As of yet, our findings support that *S. haematobium* infection and the associated bladder morbidity are significantly inter-regulated and can be addressed via MDA. On the contrary, our studied cohort in the village of Yoro suggests that *S. mansoni* infection and morbidity do not appear to be strongly dependent and as such might not be adequately tackled by simply controlling infection and egg burden via MDA. A possible confounder in such a liver lesion profile in the children of Yoro could be malaria. In fact, our study did not specifically screen for and exclude malaria cases. Understandably, the presence of this liver-affecting disease in most rural areas of Cameroon would suggest a possible and herein unappreciated role of this infection in the hepato-pathological profile of our examined schoolchildren. Larger studies on this site incorporating such a confounding disease and striving for the identification of parasite-independent factors that promote liver lesions and the onset of fibrosis during *S. mansoni* infections would therefore be needed. Such a knowledge will surely facilitate the elimination of *S. mansoni*-mediated schistosomiasis and control the *S. mansoni*-driven morbidity in a mono- or multi-infection setting.

A quite intriguing finding in our study was the negative association of *S. mansoni* infections with the robustness of serum vaccine responses. Since, we only recorded a single case of co-infection with geohelminths (*Ankylostoma spp.)* in our sub-cohort tested for measles vaccine responses, we rule out the confounding effect of geohelminths in our present analyses. *S. mansoni* infection appeared to significantly associate with lower levels of vaccine-elicited antibodies. Our findings therefore suggest that *S. mansoni* might systemically impair the host ability to mount/sustain humoral responses elicited by protective vaccines. This is not uncommon as the immunoregulation of the host vaccine responses by helminth parasites in general has been widely suggested (20, 35–37). The observed lower responses in *S. mansoni* infected children could be explained by a strong immunoregulatory cascade that has been previously described in infection with *S. mansoni* (38–40) and/or the previously reported ability of this infection to directly impair/deplete memory/vaccine responses (20, 21, 41). Further studies are clearly needed to assess causality in this observed reduction of vaccine-elicited responses in *S. mansoni* infected children and to define the mechanisms of immunoregulation involved. As of yet, our findings align with a recent report in western Kenya that demonstrated a reduced ability of children from mother diseased with schistosomiasis during pregnancy to efficiently mount an anti-measles vaccine response (19). The negative association of *S. mansoni* with anti-measles IgG antibodies in schoolchildren observed in Yoro in rural Cameroon therefore also strongly argue in favor of a more crippling burden of schistosomiasis than currently appreciated. The introduction of measles vaccination has led to longer interepidemic periods and a shift in the age distribution of remaining cases toward older children and adults (42). Our present data indicate a possible negative contribution of parasitic diseases such as schistosomiasis in lowering vaccinated children from measles vaccine-induced responses and possibly protection thus exposing them at older ages to susceptibility to measles infections/outbreaks. Of note, also, the inability of our present experimental design to dissociate between persistently infected and/or newly infected/re-infected children in our sub-cohort used for assessing anti-measles vaccine responses further indicates the distinct but herein poorly defined influence of either long term, short term or multiple term infections on children anti-measles antibody titers. Critically, however, the higher anti-measles titers in vaccinated children found not to be excreting eggs, as per our analyses, indirectly suggest a potential for MDA—which is known to change egg-positive into egg-negative individuals by means of the antiparasitic effect—in ensuring high anti-measles vaccine responses. Mechanistic studies to address the ability of *S. mansoni* infections to strip murine hosts from measles vaccine induced memory B-cell responses and the potential corrective effect of treatment with praziquantel are currently underway in our laboratory and should provide more clarification on the matter.

In conclusion, our study provides an update on the burden of schistosomiasis in two endemic sites in rural Cameroon. We emphasize the clear added value of MDA in the *S. haematobium*-infested site of Barombi Kotto but this strongly advocates for more aggressive and better tailored control strategies for the *S. mansoni*-infested site of Yoro. Our findings, although exploratory, reveal clear differences in the pathogenesis and overall burden of *S. mansoni* and *S. haematobium* that would warrant tailored approaches rather than one-works-for-all current control strategies against human schistosomiases. In as much as larger studies are further needed on the present sites, a case is sufficiently made for an added burden of schistosomiasis whereby *S. mansoni*-infected children have lower anti-measles vaccine responses.

## Author contributions

JKN: Conceptualization, Data curation, Formal analysis, Funding acquisition, Investigation, Methodology, Project administration, Resources, Supervision, Validation, Writing – original draft, Writing–review editing. SDK and PMN: Investigation, Methodology, Validation, Writing–original draft, Writing–review, and editing. SD and MS: Statistical Analyses, Methodology, Validation, Writing–original draft, Writing–review, and editing. AO: Investigation, Ultrasonography, Methodology, Validation, Writing–original draft, Writing–review, and editing. FB: Funding acquisition, Resources, Supervision, Validation, Writing–original draft, Writing–review, and editing. RM-S: Formal analysis, Funding acquisition, Investigation, Methodology, Project administration, Resources, Supervision, Validation, Writing–review and editing.

### Conflict of interest statement

The authors declare that the research was conducted in the absence of any commercial or financial relationships that could be construed as a potential conflict of interest.
